# Supplementary material for: Control of oviductal fluid flow by the G-protein coupled receptor Adgrd1 is essential for murine embryo transit
Source: Nat Commun. 2021 Feb 23;12:1251. doi: 10.1038/s41467-021-21512-w (PMC7902839; doi:10.1038/s41467-021-21512-w)
Supplement: Supplementary file 1 — Supplementary Information [file 41467_2021_21512_MOESM1_ESM.pdf]

Supplementary Information

# Control of oviductal fluid flow by the G-protein coupled receptor Adgrd1 is essential for murine embryo transit

Enrica Bianchi, Yi Sun, Alexandra Almansa-Ordonez, Michael Woods, David Goulding, Nadia Martinez-Martin and Gavin J. Wright

Correspondence to: gw2@sanger.ac.uk

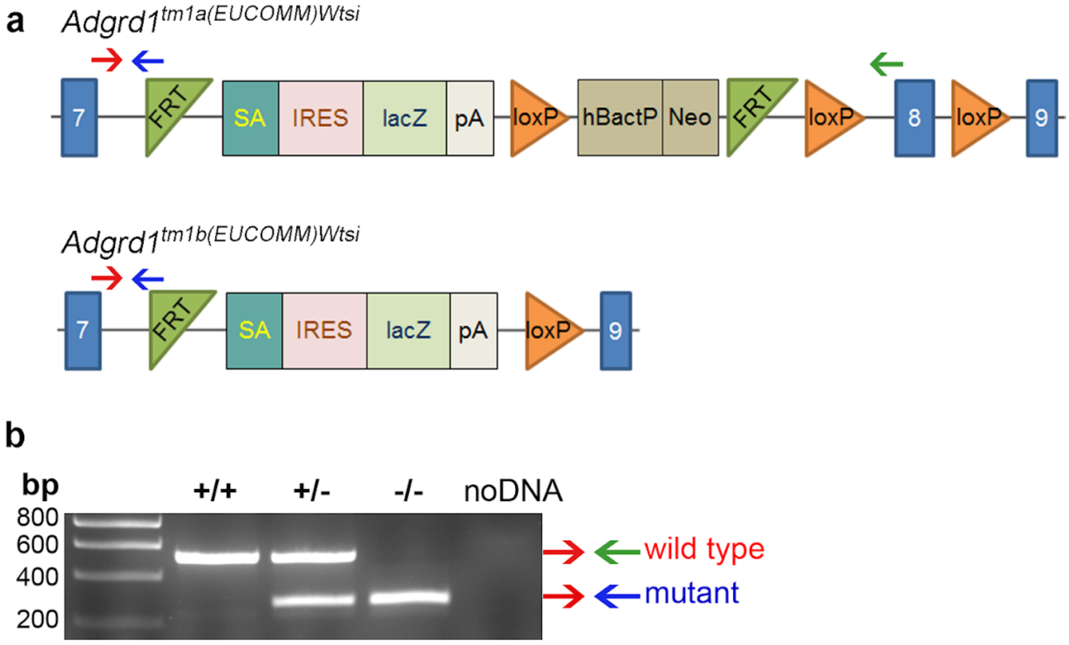


**Supplementary Fig. 1. Generation of *Adgrd1$^{-/-}$* mice using a reporter-tagged deletion allele.** **a**, Targeted ES cells carrying the "knockout-first" tm1a allele were selected using standard techniques for homologous recombination. In the tm1a allele, *Adgrd1* function is disrupted by insertion of the LacZ marker gene and a floxed Neo selection cassette upstream of exon 8. Conversion to the LacZ-tagged null allele (tm1b) lacking exon 8 was obtained by crossing *Adgrd1$^{tm1a(EUCOMM)WTSI}$* heterozygous females with *Hprt$^{Tg(CMV\text{-}Cre)Brd}$* Cre recombinase-expressing males. The colony was maintained by mating heterozygous males and females. **b**, Mice were genotyped by PCR using the indicated primers to yield products of 512bp (wild-type allele) and 263bp (mutant allele). The image is representative of more than 30 independent experiments.

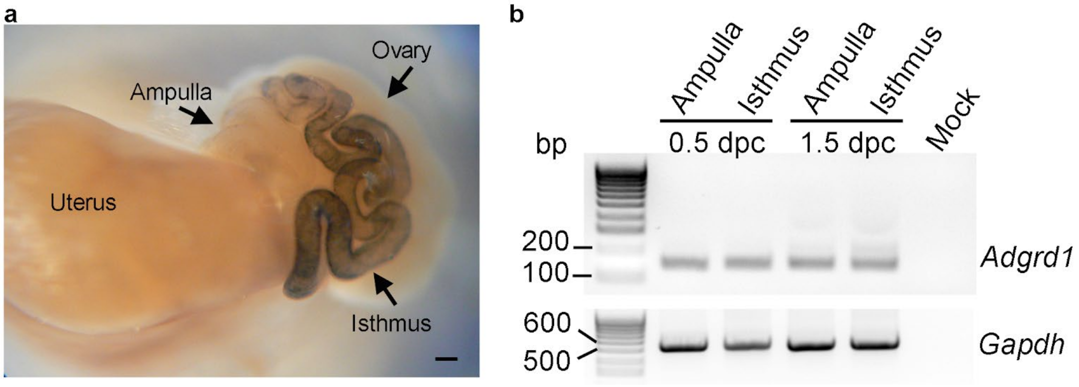


**Supplementary Fig. 2. The *Adgrd1* promoter is highly active in the oviduct and *Adgrd1* is expressed in the ampullary and isthmic epithelium at different stages of the estrus cycle. a** *Adgrd1* promoter activity in the isthmus is detected by X-gal staining of a heterozygous *Adgrd1*$^{-/+}$ female reproductive tract. Scale bar represents 200 µm. **b** Oviductal epithelial cells were collected from wild-type female mice at 0.5 dpc and 1.5 dpc. The ampulla was dissected from the isthmus, and epithelial cells were mechanically isolated from both regions before extracting RNA and performing RT-PCR. *Adgrd1* is transcribed in oviductal epithelium in both the ampulla and isthmus at similar levels at ovulation (0.5 dpc) and a day later (1.5dpc); *Gapdh* was used as a control. The image is representative of 2 independent experiments.

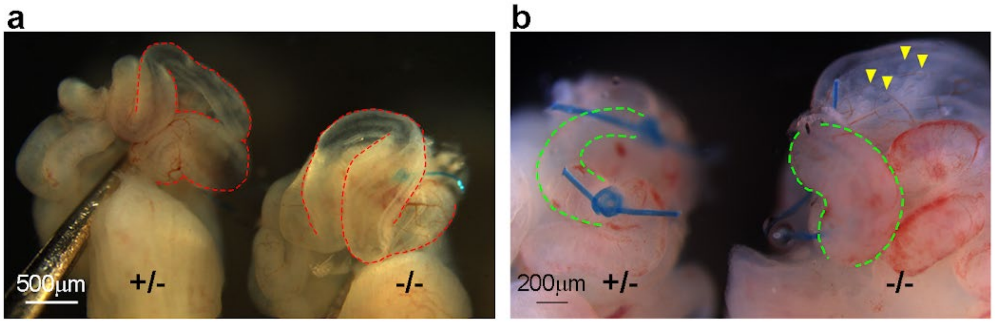


**Supplementary Fig. 3. *Adgrd1* regulates attenuation of post-ovulatory oviductal fluid production within the isthmus. a** *Adgrd1* mutant (-/-) and heterozygous control (+/-) oviducts were ligated *in vivo* at 2.5 dpc and collected four hours later; the ampullary region is highlighted by dotted red lines. A representative example of three independent experiments is shown. Homozygous mutant oviducts show a more distended ampulla compared to the heterozygous control. **b** Attenuation of oviductal fluid production is dysregulated in the isthmus of Adgrd1-mutant oviducts. Control heterozygous (left) and Adgrd1-deficient (right) oviducts at 1.5 dpc were ligated in three different locations: the infundibulum, the ampullary-isthmic junction (AIJ), and within the isthmus just after the AIJ. The oviducts were collected four hours after ligation and images collected. The dotted green line shows a larger expansion of the isthmus in the mutant oviduct compared to control. The yellow arrowheads point to oocytes ectopically located in the ampulla of Adgrd1-mutant oviduct.

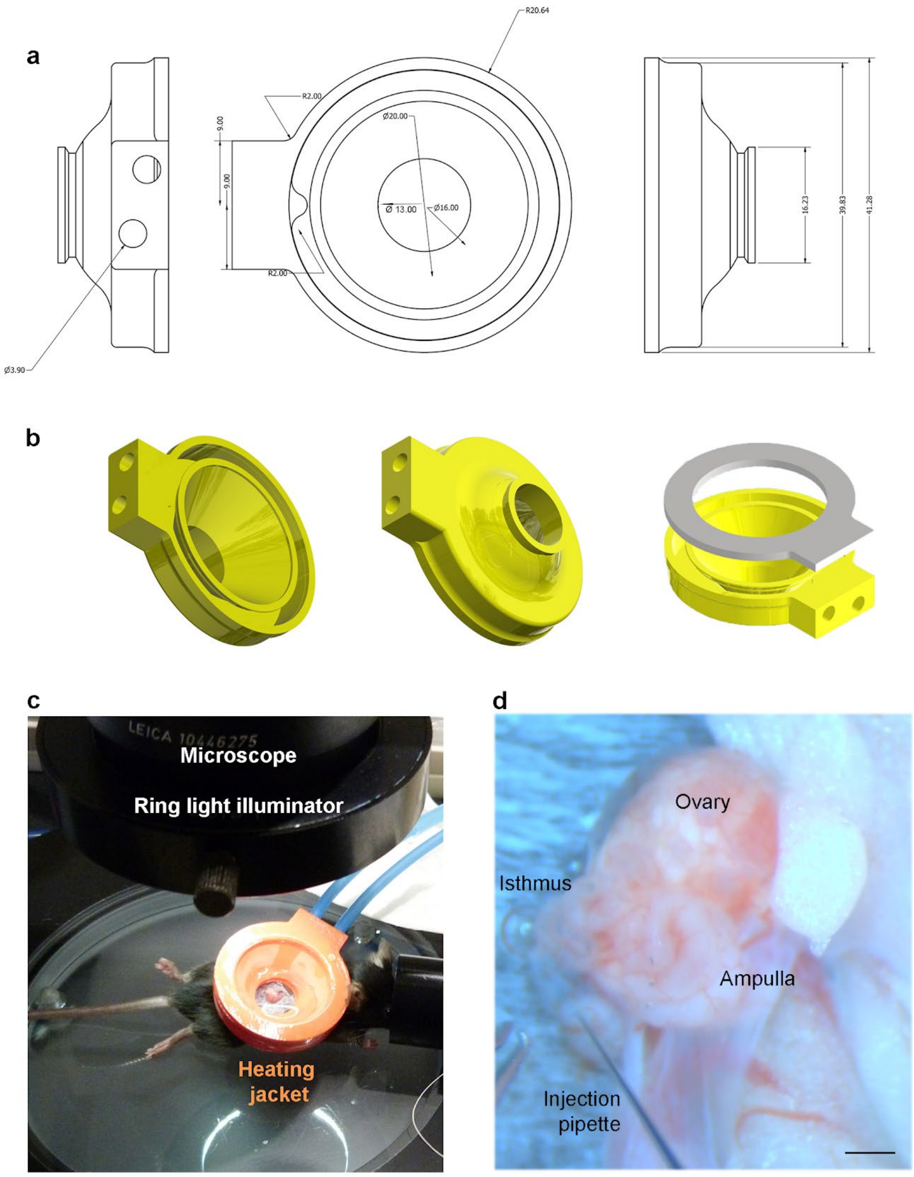


**Supplementary Fig. 4. Surgical setup to visualise oviductal fluid flow in living mice. a** A plastic heating jacket containing a continuous flow of warm water with a top diameter of 40mm and a bottom diameter of 13mm was produced by 3D printing and used to keep the buffer surrounding the exposed female reproductive tract at physiological temperature. **b** The jacket was made in two parts which were sealed with a gasket (grey) before the surface was smoothed with a Polymaker Polysher. **c** The heating jacket was connected to a motorised pump immersed in a reservoir of warm water and was applied around the surgical opening to maintain the temperature and hydration of the tissues during video recording. **d** Photo of the tracer dye being injected into the lower isthmus with a glass pipette with minimal disturbance of the neighbouring organs.

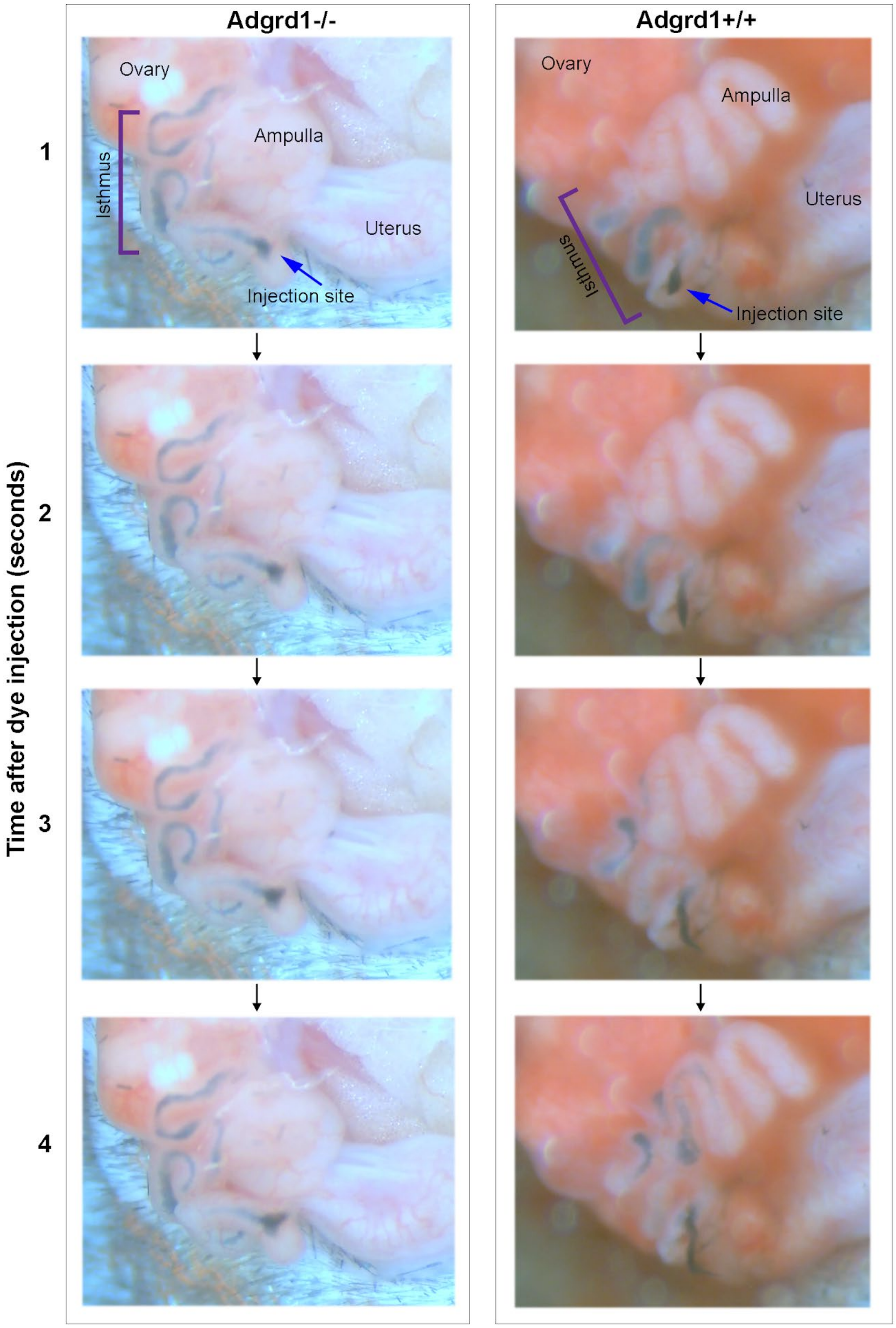


**Supplementary Fig. 5. Oviductal fluid flow is dysregulated in Adgrd1 deficient mice.** Individual frames from videos to observe the behaviour of an India ink tracer dye injected into the lower isthmus of oviducts (blue arrow) from Adgrd1-deficient (left panels) and controls (right panels); frames are separated by 1 second and start immediately following the injection of the dye. In Adgrd1-mutant mice, the tracer dye instantaneously filled the length of the oviduct as a continuous unbroken streak, whereas the dye in control oviducts segregated into discrete boluses which moved in a saltatory fashion in an adovarial direction. Images are taken from recorded videos and are representative of four mutant and five control oviducts.

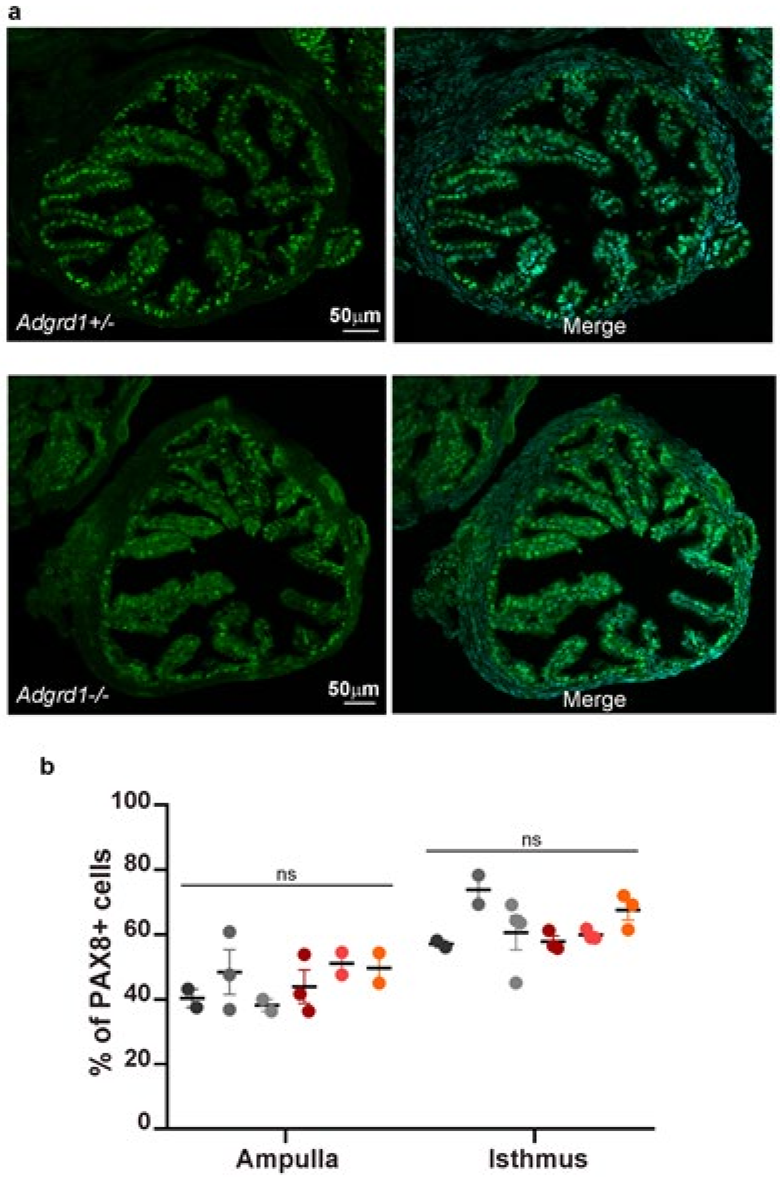


**Supplementary Fig. 6. The relative number and distribution of secretory cells appears normal in *Adgrd1*-deficient oviducts**. **a**, No overt defect is visible in the distribution of secretory cells in the epithelium of control *Adgrd*$^{+/-}$ and *Adgrd1*$^{-/-}$ oviducts. Oviductal sections from adult females in diestrous were stained with an antibody against PAX8 (green), and nuclei counterstained with DAPI (blue, merged with green in the right panels). A representative example of 3 independent experiments is shown. **b**, The number of secretory cells positively stained for PAX8 was counted in a minimum of two sections per each animal in both the ampulla and the isthmus. Each shade of colour represents a female oviduct, controls are in shades of grey and KOs are in shades of red ($n$ = 3 animals per genotype). The percentage of secretory cells is higher in the isthmus than in the ampulla in both controls and *Adgrd1*$^{-/-}$ oviducts. A two-way ANOVA analysis found that the genotype has no effect (ns) while the difference in percentage of secretory cells between the ampullary and isthmic regions of the oviduct is extremely significant ($p<0.0001$), as expected. Bars represent the mean ± SEM.

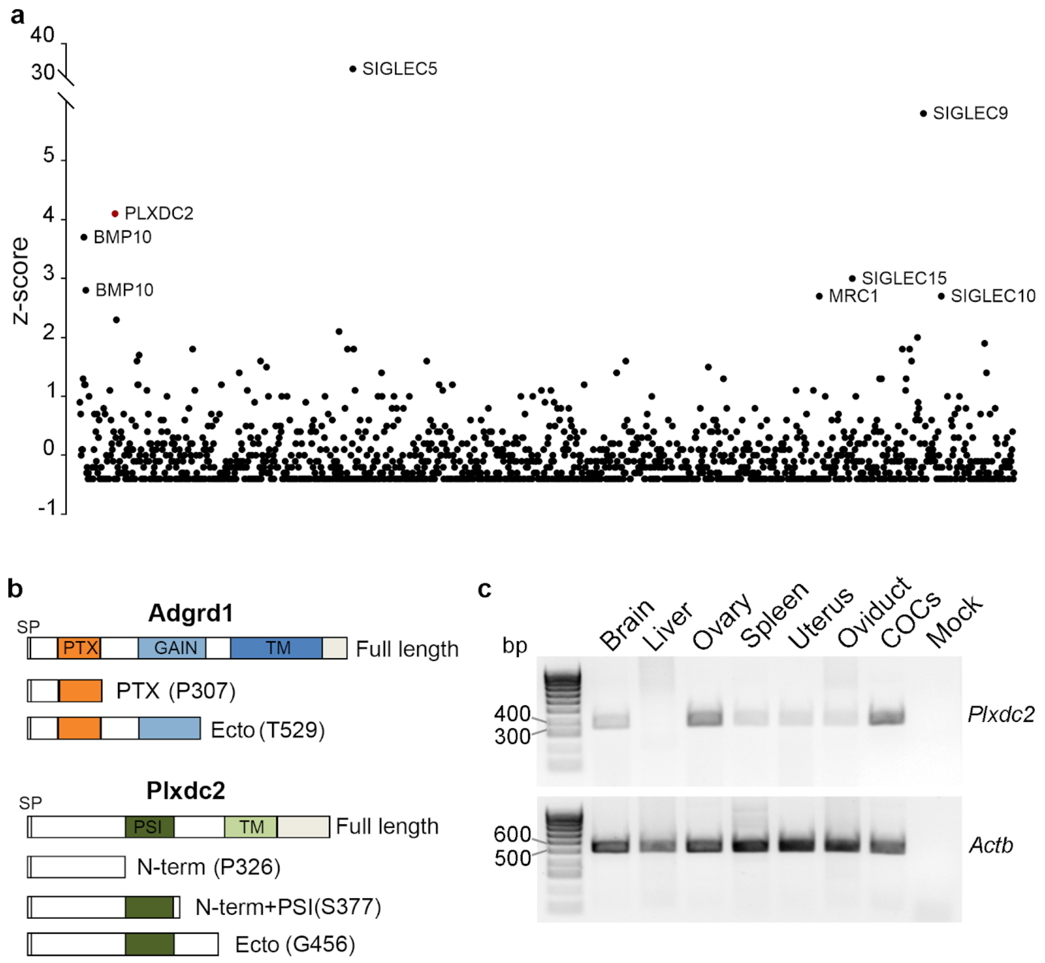


**Supplementary Fig. 7. A systematic high throughput ectodomain binding screen identified PLXDC2 as a ligand for ADGRD1. a**, A large panel of human receptor ectodomains was expressed as Fc-fusion proteins and immobilised on protein-A coated microtitre plates before testing for direct binding to a pentameric, β-lactamase-tagged ADGRD1 probe. Binding was quantified by measuring the absorbance of a hydrolysis product of β-lactamase at 485 nm. A z-score was calculated for each tested ligand and the identities of those ligands with a z-score > 2.5 are indicated, source data are provided as a Source Data file. PLXDC2 was identified as a candidate ligand due to the high z-score and by comparing ligand behaviours with other binding probes in similar screens. Members of the SIGLEC (sialic acid-binding immunoglobulin-type lectins) family and MRC1 (Macrophage mannose receptor 1) are known glycan binding proteins which are often identified in these binding screens by directly interacting with common glycans present on the protein probe. The BMP10 ligands encode secreted proteins which were also repeatedly identified in previous screens irrespective of the protein binding probe presented and so not considered further. **b**, Schematic of mouse Adgrd1 and mouse Plxdc2 constructs. SP = signal peptide; PTX = pentraxin domain; GAIN = G-protein-coupled receptor (GPCR) autoproteolysis-inducing domain; TM = predicted transmembrane region; PSI = plexin-semaphorin-integrin domain. The amino acid identities and position of the exact truncation points are reported in parenthesis. **c**, RT-PCR showing the expression of *Plxdc2* in mouse tissues and cumulus-oocyte complexes (COCs), is representative of 2 independent experiments.

**Supplementary Table 1. List of primers**

| Gene | Forward primer 5'-3' | Reverse primer 5'-3' |
|---|---|---|
| *Plxdc2* | ATCCAGGTGAAAGTCGGGTTG | GGGAGGTCGTGGTAGTTTGA |
| *Beta-actin* | ATATCGCTGCGCTGGTCGTC | AGGATGGCGTGAGGGAGAGC |
| *Adgrd1* | CCCAGGAACATCCAGGCTTT | ATTGTGGGAGAGAGGCACAG |
| *Gapdh* | AGGCCGGTGCTGAGTATGTC | TGCCTGCTTCACCACCTTCT |
| *Adgrd1*$^{tm1b(EUCOMM/Wtsi)}$ wildtype allele | ACTTTGTGGGTGGTGTCCG | CGTTCATGCAAGCCATCAC |
| *Adgrd1*$^{tm1b(EUCOMM/Wtsi)}$ mutant allele | ACTTTGTGGGTGGTGTCCG | TCGTGGTATCGTTATGCGCC |
