# Supplementary material for: Control of oviductal fluid flow by the G-protein coupled receptor Adgrd1 is essential for murine embryo transit
Source: Nat Commun. 2021 Feb 23;12:1251. doi: 10.1038/s41467-021-21512-w (PMC7902839; doi:10.1038/s41467-021-21512-w)
Supplement: Supplementary file 6 — Description of Additional Supplementary Files [file 41467_2021_21512_MOESM6_ESM.pdf]

Title: Supplementary Movie 1. Absence of Adgrd1 does not affect ciliary function on oviductal epithelium.
Description: The cilia that line the ampullary epithelium continuously beat in an abovarial direction in the Adgrd1-/- mutant oviduct. Effective transport is shown by the movement of 15 µm microparticles placed on the explant along the ampullary folds.

Title: Supplementary Movie 2. Muscle function appears normal in Adgrd1-deficient oviducts.
Description: Beads and 2-cell embryos are regularly moved back and forth due to rhythmic muscular contractions within an Adgrd1-/- ampulla explant at 1.5 dpc. A contracting segment of the isthmus is visible in the upper left corner. The video is shown at four frames per second.

Title: Supplementary Movie 3. Dysregulation of oviductal fluid flow in Adgrd1-deficient oviducts.
Description: Videos of the behaviour of a tracer dye injected into Adgrd1-deficient and control oviducts to directly observe oviductal fluid flows. Videos from the Adgrd1-mutant (left) and control (right) are shown side-by-side and have been time-matched to the start of the injection to facilitate comparison.
