# Supplementary material for: Control of oviductal fluid flow by the G-protein coupled receptor Adgrd1 is essential for murine embryo transit
Source: Nat Commun. 2021 Feb 23;12:1251. doi: 10.1038/s41467-021-21512-w (PMC7902839; doi:10.1038/s41467-021-21512-w)
Supplement: Supplementary file 7 — Reporting Summary [file 41467_2021_21512_MOESM7_ESM.pdf]

nature research

Corresponding author(s): Gavin J. Wright

Last updated by author(s): Nov 24, 2020

# Reporting Summary

Nature Research wishes to improve the reproducibility of the work that we publish. This form provides structure for consistency and transparency in reporting. For further information on Nature Research policies, see our Editorial Policies and the Editorial Policy Checklist.

## Statistics

For all statistical analyses, confirm that the following items are present in the figure legend, table legend, main text, or Methods section.

| n/a | Confirmed | |
|---|---|---|
| ☐ | ☒ | The exact sample size (*n*) for each experimental group/condition, given as a discrete number and unit of measurement |
| ☐ | ☒ | A statement on whether measurements were taken from distinct samples or whether the same sample was measured repeatedly |
| ☐ | ☒ | The statistical test(s) used AND whether they are one- or two-sided<br>*Only common tests should be described solely by name; describe more complex techniques in the Methods section.* |
| ☒ | ☐ | A description of all covariates tested |
| ☒ | ☐ | A description of any assumptions or corrections, such as tests of normality and adjustment for multiple comparisons |
| ☒ | ☐ | A full description of the statistical parameters including central tendency (e.g. means) or other basic estimates (e.g. regression coefficient) AND variation (e.g. standard deviation) or associated estimates of uncertainty (e.g. confidence intervals) |
| ☐ | ☒ | For null hypothesis testing, the test statistic (e.g. *F*, *t*, *r*) with confidence intervals, effect sizes, degrees of freedom and *P* value noted<br>*Give P values as exact values whenever suitable.* |
| ☒ | ☐ | For Bayesian analysis, information on the choice of priors and Markov chain Monte Carlo settings |
| ☒ | ☐ | For hierarchical and complex designs, identification of the appropriate level for tests and full reporting of outcomes |
| ☒ | ☐ | Estimates of effect sizes (e.g. Cohen's *d*, Pearson's *r*), indicating how they were calculated |

*Our web collection on statistics for biologists contains articles on many of the points above.*

## Software and code

Policy information about availability of computer code

Data collection: *Provide a description of all commercial, open source and custom code used to collect the data in this study, specifying the version used OR state that no software was used.*

Data analysis: *Provide a description of all commercial, open source and custom code used to analyse the data in this study, specifying the version used OR state that no software was used.*

For manuscripts utilizing custom algorithms or software that are central to the research but not yet described in published literature, software must be made available to editors and reviewers. We strongly encourage code deposition in a community repository (e.g. GitHub). See the Nature Research guidelines for submitting code & software for further information.

## Data

Policy information about availability of data

All manuscripts must include a data availability statement. This statement should provide the following information, where applicable:

- Accession codes, unique identifiers, or web links for publicly available datasets
- A list of figures that have associated raw data
- A description of any restrictions on data availability

All data generated or analysed during this study are included in this published article (and its supplementary information files) and/or available from the corresponding author on reasonable request.

# Field-specific reporting

Please select the one below that is the best fit for your research. If you are not sure, read the appropriate sections before making your selection.

- [x] Life sciences
- [ ] Behavioural & social sciences
- [ ] Ecological, evolutionary & environmental sciences

For a reference copy of the document with all sections, see nature.com/documents/nr-reporting-summary-flat.pdf

# Life sciences study design

All studies must disclose on these points even when the disclosure is negative.

| | |
|---|---|
| Sample size | No calculation was done to predetermine the sample size as the effect size was large, infertility in the knockout mice is 100% penetrant. |
| Data exclusions | No data were excluded from the analyses |
| Replication | All attempts at replication were successful |
| Randomization | Allocation of mice was not randomized. Animals were allocated based on their genotype. |
| Blinding | Blinding was not possible because the knockout phenotype is clearly visible, eggs are found in the upper tract of the oviduct at any time. |

# Reporting for specific materials, systems and methods

We require information from authors about some types of materials, experimental systems and methods used in many studies. Here, indicate whether each material, system or method listed is relevant to your study. If you are not sure if a list item applies to your research, read the appropriate section before selecting a response.

## Materials & experimental systems

| n/a | Involved in the study | |
|---|---|---|
| [ ] | [x] | Antibodies |
| [ ] | [x] | Eukaryotic cell lines |
| [x] | [ ] | Palaeontology and archaeology |
| [ ] | [x] | Animals and other organisms |
| [x] | [ ] | Human research participants |
| [x] | [ ] | Clinical data |
| [x] | [ ] | Dual use research of concern |

## Methods

| n/a | Involved in the study | |
|---|---|---|
| [x] | [ ] | ChIP-seq |
| [x] | [ ] | Flow cytometry |
| [x] | [ ] | MRI-based neuroimaging |

## Antibodies

| | |
|---|---|
| Antibodies used | Polyclonal rabbit-anti-mouse Adgrd1 (Cambridge Research Biochemicals); polyclonal rabbit-anti-mouse Plxdc2 (Cambridge Research Biochemicals); rat monoclonal anti-mouse Juno (clone TH6, Biolegend); mouse monoclonal anti-acetylated tubulin (clone 6-11B-1, Sigma Aldrich); mouse monoclonal anti-Flag-Cy3 conjugate (clone M2, Sigma Aldrich); Polyclonal rabbit anti-beta-actin (Abcam, ab8227); anti- Pax8 rabbit polyclonal antibody (10336-1-AP, Proteintech); monoclonal anti-alpha smooth muscle Actin (clone 1A4, A2547, Sigma); and Texas-red conjugated phalloidin (Thermo Fisher, T7471). Ox68 monoclonal antibody, Horseradish peroxidase (HRP)-conjugated anti-rabbit antibody (Life technologies), Alexa Fluor 488 goat anti-mouse (Thermo Fisher), Alexa Fluor 647 goat anti-rabbit (Jackson Immunoresearch) |
| Validation | Agdrd1 and Plxdc2 Polyclonals were validated by Western Blot on recombinant proteins and by immunofluorescence on transfected HEK293T cells. Anti-Juno, Ox68 and anti-Flag-Cy3 were used in a previous publication (PMID:24739963). Anti-Acetylated tubulin, Pax 8, anti-alpha smooth muscle Actin, and anti-Actin were validated by the manufacturers and used in peer-reviewed papers. |

## Eukaryotic cell lines

Policy information about cell lines

| | |
|---|---|
| Cell line source(s) | HEK293T were obtained from ATCC, HEK293-6E were a kind gift of Professor Yves Durocher (PMID:18752669) |
| Authentication | None of the cell lines used were authenticated. |
| Mycoplasma contamination | HEK293-6E cells tested negative for mycoplasma contamination. HEK293T cells were not tested for Mycoplasma. |
| Commonly misidentified lines (See ICLAC register) | *Name any commonly misidentified cell lines used in the study and provide a rationale for their use.* |

## Animals and other organisms

Policy information about studies involving animals; ARRIVE guidelines recommended for reporting animal research

| | |
|---|---|
| Laboratory animals | Laboratory animals used are of the species Mus musculus, C57BL/6N strain. Sex and age are specified in the main text and in the methods. |
| Wild animals | *Provide details on animals observed in or captured in the field; report species, sex and age where possible. Describe how animals were caught and transported and what happened to captive animals after the study (if killed, explain why and describe method; if released, say where and when) OR state that the study did not involve wild animals.* |
| Field-collected samples | *For laboratory work with field-collected samples, describe all relevant parameters such as housing, maintenance, temperature, photoperiod and end-of-experiment protocol OR state that the study did not involve samples collected from the field.* |
| Ethics oversight | Research was approved by the Sanger Institute Animal Welfare and Ethical Review Board. All animal experiments were performed under UK Home Office governmental regulations and European directive 2010/63/EU. |

Note that full information on the approval of the study protocol must also be provided in the manuscript.
